# Registrations of Assistance Dogs in California for Identification Tags: 1999–2012

**DOI:** 10.1371/journal.pone.0132820

**Published:** 2015-08-19

**Authors:** Mariko Yamamoto, Mayllynne T. Lopez, Lynette A. Hart

**Affiliations:** School of Veterinary Medicine, University of California Davis, Davis, California, United States of America; Faculty of Animal Sciences and Food Engineering, University of São Paulo, Pirassununga, SP, Brazil, BRAZIL

## Abstract

Dogs are filling a growing number of roles supporting people with various disabilities, leading to a chaotic situation in the U.S. Although the federal laws allow public access with working dogs only for people with disabilities, no governmental enforcement or management system for such dogs exists. Furthermore, there is no substantive way to confirm whether the dog is an adequately trained assistance dog or not, as neither the handlers nor the dogs are required to carry any particular certification or identification. Therefore, unqualified assistance dogs and incidents such as dog bites by assistance dogs sometimes are problems in the U.S. A governmental oversight system could reduce problems, but no information is available about the current uses of assistance dogs in the U.S. We aimed to investigate the current demographics of registered assistance dogs and the evolving patterns in uses of dogs during 1999–2012 in California. We acquired data on assistance dogs registered by animal control facilities throughout California. We used descriptive statistics to describe the uses of these assistance dogs. The number of assistance dogs sharply increased, especially service dogs, in the past decade. Dogs with small body sizes, and new types of service dogs, such as service dogs for psychiatric and medical assistance, strongly contributed to the increase. The Assistance Dog Identification tags sometimes were mistakenly issued to dogs not fitting the definition of assistance dogs under the law, such as emotional support animals and some cats; this reveals errors in the California governmental registering system. Seemingly inappropriate dogs also were registered, such as those registered for the first time at older than 10 years of age. This study reveals a prevalence of misuse and misunderstanding of regulations and legislation on assistance dogs in California.

## Introduction

At the early domestication of dogs, humans and wolves presumably came into contact regularly; both were social animals and hunted many of the same prey items [1], cooperatively and efficiently [2]. From that time, dogs have continued to be very close to humans. For example, paintings and literature from as early as 79 CE show that dogs were used as guides for blind persons [3]. Systematic training for guide dogs did not start until World War I, but even earlier, people with visual disabilities who lived with their dogs would have naturally known that dogs could help them to walk outside. In a similar way with hearing dogs, the first hearing dog was trained at the request of a woman with hearing disabilities [4]. Her previous dog had instinctively learned to detect sounds for her; after her dog died she sought out a person who could train a dog for her. These examples reveal that the initial idea of using dogs as assistants for humans with disabilities undoubtedly arose spontaneously through the long history of human-dog interaction [3, 4].

The first guide dog school was established in Nashville, Tennessee, in the United States in 1929. By now, dogs perform many tasks for assisting people with disabilities; these have been conceived and tailored to their handlers’ specific needs. These dogs are valuable for the people with disabilities living with the dogs, as well as for their family members and others interacting with the persons. Some studies have shown that assistance dogs bring physical, psychological, and social effects for their handlers [5–10]. A primary direct benefit from the dogs is the increased independence which leads to handlers leaving the house more frequently and a reduced requirement for paid and unpaid assistance [5, 7–10]. Increased social interactions and psychological benefits from the dogs, such as increased self-esteem and confidence, and decreased anxiety and stress [5–10] may also relate to the positive effects mentioned above. As these studies show, the expectations for the assistance dogs are high and expanding. The status of these dogs in society has changed dramatically in the past few decades, leading to new laws and regulations to protect the rights of people with disabilities by allowing them reasonable access with their dogs.

### Definitions and taxonomies of assistance dogs in laws and regulations in the U.S. and other countries

In the U.S., handlers with disabilities are allowed to bring their assistance dogs wherever they go, but not their pet dogs nor even therapy dogs. However, the U.S. has no process for concrete registration for these dogs. “Service animal” or “service dog” is the term used for these dogs in the U.S. federal laws [11], but various terms are used. An international advocacy organization, Assistance Dogs International (ADI) defines “assistance dogs” as including “guide dogs” for people with visual disabilities, “hearing dogs” for people with hearing disabilities, and “service dogs” for people with all disabilities except visual or hearing [12]; the state of California also uses this nomenclature. However, the taxonomies of these dogs assisting their handlers with disabilities are inconsistent throughout the world as well as the U.S. For example, Queensland, Australia, uses “guide dogs, hearing dogs, and assistance dogs” [13], and New Zealand uses “Disability Assist Dogs” as a general term for these dogs [14]. On the other hand, “assistance dogs” is used as a general term under the regulations in the EU, including the U.K., similar to ADI [15, 16]. However, the EU and U.K. sometimes separate “guide dogs” from other types of assistance dogs and describe them as “guide dogs and other assistance dogs (hearing dogs and service dogs)” [17]. Therefore, these terms need to be specified to avoid confusion. Here, we used data for assistance dogs registered in California, and the definition of assistance dogs under the California Food and Agriculture Code is similar to the definition by ADI. Therefore, we use the definition and taxonomy of assistance dogs from ADI: “assistance dogs” is the general term for dogs supporting their handlers with disabilities, which consist of “guide dogs”, “hearing dogs”, and “service dogs”.

To assure equal or reasonable accommodation and prevent discrimination against people with disabilities, some U.S. federal laws and regulations protect their rights to be accompanied by their assistance dogs in public settings. Relevant legislation has been issued by the U.S. Department of Justice (DOJ: 2010, 2011, [18]), the U.S. Department of Transportation (DOT: 2008, [19]), and the U.S. Department of Housing and Urban Development (HUD: 2008, [20]). These three agencies oversee three domains: public accommodations, including but not limited to restaurants, hospitals, hotels, and shopping centers; public transport; and housing settings, respectively. DOJ (2011) has the most extensive regulations, building on the Americans with Disabilities Act (ADA), and has defined assistance dogs (termed “service animals” and “service dogs”) as individually trained animals that perform tasks for the benefit of individuals with disabilities; only dogs and miniature horses are included since this updated regulation [18]. In contrast, DOT and HUD use a broader definition for assistance dogs, and include emotional support animals (ESAs) for reasonable accommodation, including dogs, as required in the ADA [19, 20]. ESAs reduce stress or anxiety of their handlers, who can be accompanied by such dogs. A letter from a doctor to verify the person’s needs for the animal may be requested, and appropriate behavior of the dog is required. ESAs differ from the assistance dogs described by DOJ (2011) as “service dogs”, in that no special training in tasks is required for the dog; these abilities to comfort and provide a nexus with the person’s disabilities are considered innate and sufficient. Any type of animal except unusual animals, which could pose unavoidable safety and/or public health concerns, such as snakes, other reptiles, ferrets, rodents, and spiders, is within the definition of ESAs [19].

### Assistance dogs in the public

The laws and regulations mentioned above do not specify a means of enforcement to assure appropriate practice with assistance dogs, such as methods or venues for their training, or qualification or registration of those dogs. Also, the definition of disabilities is very broad in the U.S. [21], and people are allowed to create different tasks for the dog to accommodate their disabilities in various aspects of their life. While many people claim their dogs as assistance dogs, there is no way to know the number or quality of assistance dogs working in the U.S., nor to distinguish a proficient assistance dog from an unqualified one. Lacking enforcement, some unfortunate incidents involving assistance dogs have occurred, including death and serious injuries [22, 23]; these tragedies have led to modifications of policies for allowing and subsidizing assistance dogs for soldiers and veterans [24, 25].

In the U.S. Army, assistance dogs are allowed or subsidized only for people who are approved by the multidisciplinary department team; dogs must be acquired through organizations accredited by Assistance Dogs International (ADI). Similarly, the Veterans’ Administration offers subsidies only for dogs trained and placed by an ADI accredited facility. Since policies affecting soldiers and veterans became more stringent, psychiatric service dogs that would support someone with post traumatic stress disorder (PTSD) or anxiety have been excluded by the U.S. Army and Veterans’ Administration, as well as service dogs not trained by an ADI-accredited facility. The stated reason for excluding the psychiatric service dogs is that there was insufficient evidence of their beneficial impact for their handlers.

Outside the U.S., public access for people with disabilities accompanying their assistance dogs is protected in many countries in Europe, Australia, New Zealand, and some Asian countries. Unlike the U.S., these countries tend to limit the assistance dogs only to those which were certified by specified training organizations and require the assistance dog handlers to carry an identity card and put an identifying harness or coat on their dogs [13–15, 26].

### California assistance dog ID tags

Assistance dog identification tags (ID tags) are issued in California for handlers or trainers of assistance dogs, usually by the local animal control department or county clerk [27]. The dog licensing fee is waived for dogs receiving these ID tags. The Code requires dogs in training to be tagged with the ID tags when they are taken to public accommodations, but this is not mandatory for active assistance dogs. The applicants provide information regarding their dogs, such as breed, gender, type of assistance, and tasks the dogs perform, and sign an affidavit of understanding the Penal Code prohibiting any fraud. This information is available to the public under the Freedom of Information Act. Under the California Food and Agriculture Code, “assistance dogs” comprise dogs specially trained as guide dogs, signal dogs, or service dogs (§30850 (a)). Types of assistance dogs were defined as: “guide dogs” trained by a person licensed under California Business and Professions Code [28] or as defined in the ADA; “signal dogs” (or hearing dogs) trained to alert an individual who is deaf or hearing impaired to intruders or sounds; “service dogs” individually trained to the requirements of the individual with a disability, including, but not limited to, minimal protection work, rescue work, pulling a wheelchair, or fetching dropped items (§54.1 (b)) [29]. Concerning ‘minimal protection’, the revised ADA [18] modified it to ‘non-violent protection’ since it could be interpreted to allow any dog that is trained to be aggressive to qualify as a service animal (dog) simply by pairing the animal with a person with a disability. But in California, ‘minimal protection work’ language is still used in the Code.

The information provided for the issued California tags does not reflect all working assistance dogs in the state. However, with no mandatory registration system for assistance dogs in the U.S., these California registrations offer a unique view of the demographics and specific roles of assistance dogs in California and may help to clarify sources of some of the challenges with assistance dogs in the U.S. We aimed to investigate the current demographics of registered assistance dogs in California, and to study the trends of registered assistance dogs over time to understand evolving patterns in uses of assistance dogs. For this study, we requested and acquired the available data from the animal control facilities throughout California.

## Materials and Methods

### Data collection

To obtain the available information on registrations, we contacted all 290 animal control facilities listed in the California Animal Control Directors Association by telephone or email. When an animal control facility did not issue tags, we requested a referral to the facility, such as the county clerk’s office, which issued the tags in that area. For facilities that did not respond, we attempted contact again after an interval of 1 to 2-weeks; those with no answer after three attempts were not included in the study. We requested all available information in the registration record except any personal information pertaining to the owner of the dog. Since the data were redacted to remove personal identifiers, an IRB protocol was not required. The information was acquired in whatever manner was convenient for the facility, through telephone, email, fax, or mail. All data were collected between January and June 2013; information on tags issued up through 2012 was used in the analyses.

### Types of data and categories

The provided information included the ages, genders, and breeds of dogs, categories of assistance dogs, including guide, hearing (signal), and service dogs, details of assisting tasks, and the dates of registration. However, some details differed in the information provided by the various animal control facilities. We grouped the available data according to specific research questions.

The animal control facilities used a variety of data storage formats: I. maintaining data storage for all registered dogs over time, whether or not the dogs were still working (dataset I); II. storing data on dogs actively working at the time data were provided to us (dataset II); III. storing data for all registered dogs, whether or not the dogs were still working. The duration of data storage varied among the animal control facilities; most facilities purged records after a specified period of time, e.g., storage could be for 5, 7, or 10 years (dataset III). Each dog was counted only once. To investigate the changes in breeds, body sizes, and tasks of dogs from 1999 through 2012, only dataset I was used.

### Types of assistance dogs

We categorized three types of assistance dogs: guide dogs, hearing dogs, and service dogs, as defined by the ADI. We classified each dog’s record based on data from the animal control facilities pertaining either to the type of assistance dog and/or the detailed tasks performed by the dog. For service dogs with detailed tasks listed, we further categorized them into sub-categories of working roles, including service dogs for mobility, psychiatric, and medical assistance, emotional support animals, and others. Although emotional support animals are not recognized as service animals (assistance dogs) under the U.S. DOJ, nor the Food and Agriculture Code in California, many emotional support animals had been registered as service dogs, revealing the limited understanding of the correct definition of assistance dogs. Therefore we included the registered emotional support dogs as service dogs in the analyses, as further clarified in the discussion. Dogs were categorized only when a clear description was provided; data with ambiguous descriptions were excluded, e.g., “alerting medical problem” could be describing either responses to medical or psychiatric symptoms. The category of emotional support dogs was used only for dogs simply described as for “emotional support”; dogs supporting psychiatric disabilities were categorized as psychiatric service dogs.

### Sizes of dogs

Generally the information did not provide details on the sizes or weights of dogs, but most dogs were listed as specific pure breeds or mixed breed types. To assess the sizes of registered dogs, we analyzed only dogs listed as purebred dogs or identified mixed breeds for which parents’ breeds were categorized into a single size group. We used these listed purebreds or the mixed breeds where parents of similar size were described to categorize these dogs into five groups according to their heights as listed by the American Kennel Club [30], or the Dog Breed Info Center [31] in cases where the AKC did not list heights. We determined the published median heights for each breed using their size ranges. These median heights were classified as five size groups: 1. less than 11 inches (e.g., Chihuahua and Yorkshire Terrier); 2. 11 inches or more but less than 16 inches (e.g., American Cocker Spaniel and Jack Russell Terrier); 3. 16 inches or more but less than 21 inches (e.g., Australian Shepherd and Border Collie); 4. 21 inches or more but less than 26 inches (e.g., Labrador Retriever and German Shepherd); 5. 26 inches or more (e.g., Doberman Pinscher and Great Dane). Then, we further consolidated these groups into three categories: small: sizes 1 and 2; medium: size 3; and large: sizes 4 and 5. The dogs that were mixed breeds where parents were of different size categories or unspecified or unknown breeds were not included in the analyses related to the sizes of dogs. However, as an exception, although Pit Bull is a mixed breed type rather than purebred, we included Pit Bull in size 3 according to the Dog Breed Info Center, because many Pit Bulls were claimed as assistance dogs.**Statistical analysis and data management**

We used a descriptive research design, employing descriptive statistics to investigate our research questions: 1. the types of assistance for the registered assistance dogs (guide, hearing, and service dogs), 2. their categories of work (e.g., guide dogs, hearing dogs, service dogs for mobility, psychiatric, and medical assistance), 3. the detailed tasks they performed, 4. their breeds, body sizes, and ages, 5. the relations of the categories of work and their body sizes, and 6. the patterns of change over the last decade for the items mentioned above. Table 1 shows the data management for each research question. Dataset 1 was used to investigate the research questions related to changes over years 1999–2012; for the other questions we used all datasets. Because the types of information collected by the animal control facilities somewhat varied, the number of dogs in each denominator for each research question varied.

**Table 1:** The datasets and the items of data used for each research question.

Research Questions	Dataset	Items
Entire Data		
Types of assistance	Datasets1-3	Types of assistance (optional): guide dog, hearing dog, service dog
Categories of work	Datasets 1–3	Description of tasks indicated by the applicants
Detailed tasks performed by dogs	Datasets 1–3	Description of tasks
Body sizes of dogs	Datasets 1–3	Breeds
Breeds	Datasets 1–3	Breeds
Ages + body sizes	Datasets 1–3	Ages, breeds
Categories of work + body sizes	Datasets 1–3	Descriptions of tasks, breeds
Categories of work + breeds	Datasets 1–3	Descriptions of tasks, breeds
Detailed tasks + body sizes	Datasets 1–3	Descriptions of tasks, breeds
Changes over Years		
Number of registered dogs	Dataset 1	Date of registration
Types of assistance for each year	Dataset 1	Date of registration, types of assistance
Categories of work for each year	Dataset 1	Date of registration, description of tasks
Breeds for each year	Dataset 1	Date of registration, breeds
Body sizes for each year	Dataset 1	Date of registration, breeds
Body sizes + categories of work for each year	Dataset 1	Date of registration, breeds, description of tasks

## Results

**Fig 1:**
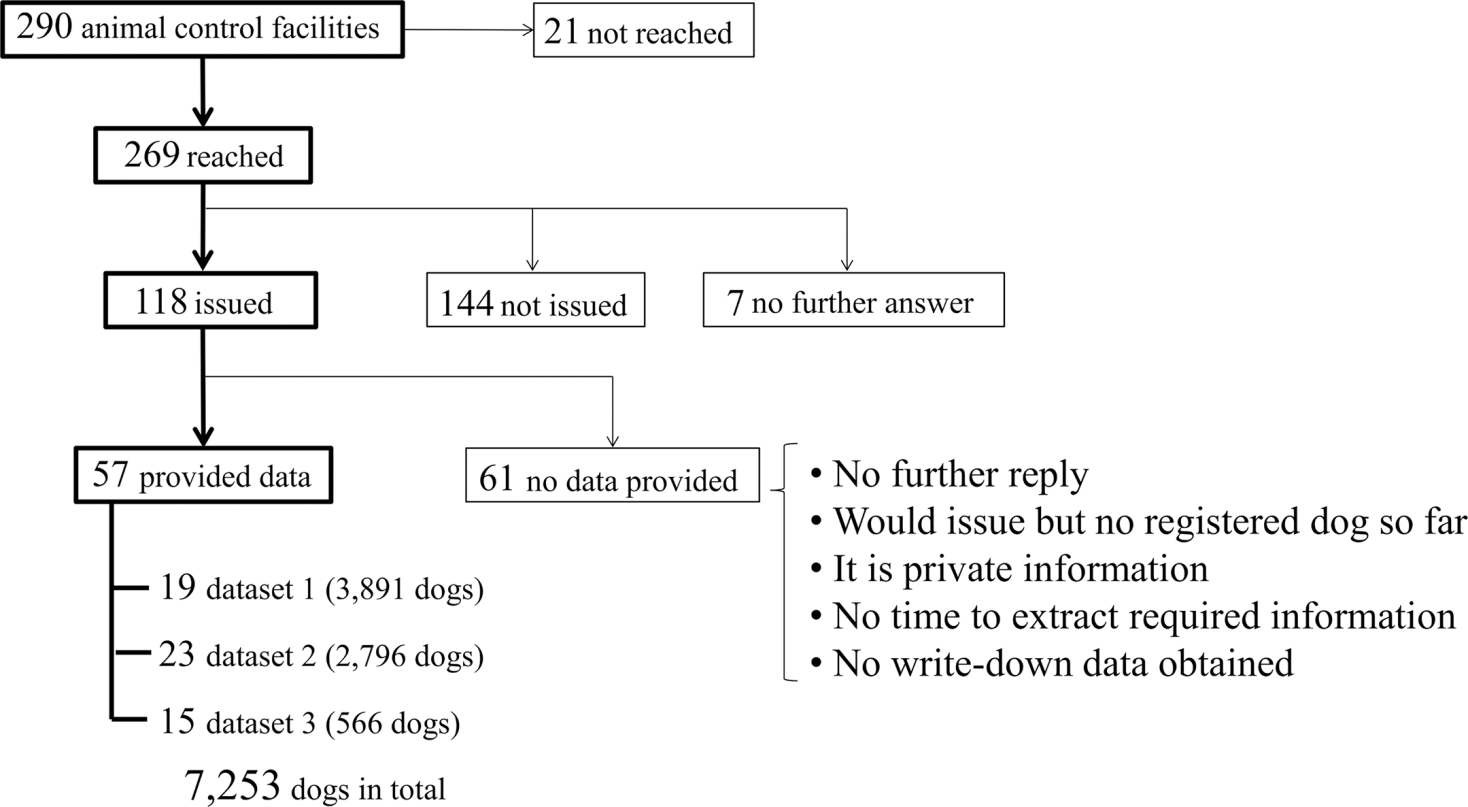
The responses of animal control facilities and the numbers of facilities and registered dogs in each dataset.

Among 290 animal control facilities listed in the California Animal Control Directors Association, 57 facilities provided the data concerning 7,253 dogs registered during 1999 through 2012. Fig 1 shows the number of facilities which issued the ID tags and categorizes their responses to this research, also indicating the number of facilities and registered dogs for each dataset. Large populated counties or cities such as San Francisco, Los Angeles, and San Diego accounted for a large proportion of the registrations.

### Registrations of all dogs by types of assistance, categories of work, detailed tasks, and body sizes

Tables 2 and 3 summarize the results for the research questions.

**Table 2:** Summary for the profiles of the data: types of assistance, body sizes, breeds, and ages.

Research questions	Number of dogs	Details	n
		Guide dogs	168
		Hearing dogs	114
**Types of assistance**	2,998	Service dogs	2,599
		Dogs performing multiple tasks	80
		Non-assistance dogs	37
		Small	2,563
**Body sizes**	5,347	Medium	583
		Large	2,226
		Labrador Retriever	688
		Chihuahua	499
		German Shepherd Dog	313
		Golden Retriever	300
		Yorkshire Terrier	209
		Shih Tzu	134
		Pomeranian	133
		Pit Bull	130
		Dachshund	128
**Purebred**	4,937	Maltese	106
		Standard Poodle	99
		Miniature Poodle	96
		Australian Shepherd	82
		Pug	65
		Toy Poodle	64
		Rottweiler	64
		Border Collie	62
		Bichon Frise	61
		Jack Russell Terrier	61
		Boston Terrier	58
		< 1 year old	902
		1–2 years old	1,700
**Ages**	6,024	3–4 years old	1,292
		5–6 years old	859
		7–9 years old	793
		10 years old	478

**Table 3:** Body sizes and ages of dogs and their categories of work.

Research questions	Number of dogs			%	
		Age (years)	Small	Medium	Large
		< 1 (n = 720)	36.7	11.0	52.4
**Body sizes and ages**		1–2 (n 1,275)	47.3	11.0	41.7
**(years)**	4,528	3–4 (n = 948)	50.5	10.3	39.1
		5–6 (n = 659)	52.7	11.1	36.3
		7–9 (n = 581)	49.4	11.0	39.6
		> 10 (n = 345)	60.9	13.9	25.2
		Working role	Small	Medium	Large
		Guide (n = 168)	5.5	5.5	89.0
		Hearing (n = 72)	54.2	11.1	34.7
**Body sizes and**		Mobility (n = 154)	12.3	10.4	77.3
**categories of work**	786	Psychiatric (n = 103)	51.5	20.4	28.2
		Medical (n = 43)	34.9	9.3	55.8
		Emotional (n = 43)	65.1	7.0	27.9
		Multiple (n = 208)	31.3	13.9	54.8

#### Types of assistance dogs and categories of working roles among registered dogs

Among all the registered dogs, classifications for the types of assistance were available for 2,998 dogs; a strong majority of these were service dogs. There were also 37 registered animals (mainly dogs and a few cats) which were not legitimate assistance dogs under the law, such as police dogs, therapy dogs, search and rescue dogs, dogs released from assistance dog programs, and companion dogs. These were excluded from the study despite having been registered.

Among the 2,599 service dogs, the categories of work were available for 768 dogs. Among service dogs, use for mobility assistance was the most common category, and psychiatric support was the second. Dogs were also often registered for emotional disabilities.

#### Body sizes and breeds of registered dogs

The number of dogs with small body sizes exceeded the number with large body sizes. Among the 4,937 purebred dogs, the most commonly registered breed was Labrador Retriever, and Chihuahua was second most common. The registration frequency of various breeds is described in Table 2. Also, 6 tags were issued for cats.

#### Ages of dogs at the first registration and their body sizes

Ages at the first registration were available for 6,024 dogs. Younger dogs were registered more often than older dogs (10 years old or more) but a substantial number of older dogs were registered as assistance dogs. Among these dogs, body sizes were obtained for 4,528 dogs. These older dogs that were registered were more likely to have smaller body sizes.

#### Categories of working roles as related to body sizes or breeds

Both the categories of work and the body sizes were obtained for 786 dogs. Large dogs were common among guide dogs and service dogs used for mobility assistance, while small dogs were common among hearing dogs, psychiatric service dogs, and emotional support dogs.

Among pure breeds, categories of work were available for 675 dogs (guide: 126 dogs; hearing: 65; mobility: 136; psychiatric: 90; medical: 37; emotional: 34; multiple: 187). Table 4 shows the number of pure breeds and the most commonly used breed for each category of work.

**Table 4:** The number of pure breeds registered and the most commonly used breed for each category of work.

Category of work	Number of pure breeds	Most common breed(number of dogs)
**Guide dogs**	**15**	**Labrador Retriever (89)**
**Hearing dogs**	**26**	**Chihuahua (10)**
**Service dogs for**	****	****
**Mobility**	**46**	**Labrador Retriever (31)**
**Psychiatric**	**42**	**Chihuahua (9)**
**Medical**	**19**	**Labrador Retriever (9)**
**Emotional**	**18**	**Chihuahua, Labrador (5 for each)**
**Multiple**	**57**	**German Shepherd Dog,Labrador Retriever (22 for each)**

#### Specific tasks and the dogs’ body sizes

The various roles of service dogs accommodated people with a range of disabilities other than hearing and visual. Thus, the tasks performed by service dogs were more personalized compared to guide dogs and hearing dogs. Tables 5, 6 and 7 show the specific tasks of mobility, psychiatric, and medical service dogs and their body sizes. These tables include service dogs which performed multiple categories of work.

**Table 5:** Numbers of service dogs performing specific tasks for mobility support [%].

	Size			
	Small	Medium	Large	Total
**Specific tasks**	n [%]	n [%]	n [%]	n [%]
Fetch items/retrieve, carry item to others	27 [75.0]	19 [57.6]	133 [74.3]	179 [72.2]
Balance	8 [22.2]	13 [39.4]	64 [35.8]	85 [34.3]
Open/close doors	4 [11.1]	5 [15.2]	48 [26.8]	57 [23.0]
Carry objects	2 [5.6]	7 [21.2]	28 [15.6]	37 [14.9]
Pull/push wheelchair	2 [5.6]	6 [18.2]	27 [15.1]	35 [14.1]
Turn lights/switch on/off	0 [0.0]	1 [3.0]	29 [16.2]	30 [12.1]
Support to stand up or sit down/getting	1 [2.8]	4 [12.1]	24 [13.4]	29 [11.7]
out of the bed				
Emergency response	4 [11.1]	1 [3.0]	8 [4.5]	13 [5.2]
(get help, bring phone, call 911)				
Help undress/dress	1 [2.8]	0 [0.0]	4 [2.2]	5 [2.0]
Elevator button	1 [2.8]	0 [0.0]	2 [1.1]	3 [1.2]
Remove/cover blanket	0 [0.0]	0 [0.0]	2 [1.1]	2 [0.8]
Transfer from one place to another	0 [0.0]	0 [0.0]	2 [1.1]	2 [0.8]
Unlock door	0 [0.0]	0 [0.0]	1 [0.6]	1 [0.4]
Total number of dogs	36	33	179	248

**Table 6:** Numbers of service dogs performing specific tasks for psychiatric support [%].

	Size			
	Small	Medium	Large	Total
**Specific tasks**	n [%]	n [%]	n [%]	n [%]
Calm/alleviate anxiety/depression	22 [55.0]	9 [42.9]	11 [29.7]	42 [42.9]
Alert panic attack/mood	12 [30.0]	5 [23.8]	14 [27.8]	31 [31.6]
Calm/stay during panic attack or when upset	4 [10.0]	4 [19.0]	2 [5.4]	10 [10.2]
Alert someone at door/intruder	6 [15.0]	0 [0.0]	3 [8.1]	9 [9.2]
Relieve stress	5 [12.5]	1 [4.8]	1 [2.7]	7 [7.1]
Tactile stimuli/deep pressure	2 [5.0]	1 [4.8]	4 [10.8]	7 [7.1]
Medication reminder/bring medication	2 [5.0]	1 [4.8]	4 [10.8]	7 [7.1]
Area assessment	1 [2.5]	0 [0.0]	5 [13.5]	6 [6.1]
Buffer between people (boundary)	1 [2.5]	3 [14.3]	2 [5.4]	6 [6.1]
Interrupt destructive or repetitive behavior	0 [0.0]	1 [4.8]	4 [10.8]	6 [6.1]
Protection	0 [0.0]	1 [4.8]	4 [10.8]	5 [5.1]
Change what owner is thinking about/mood	2 [5.0]	0 [0.0]	2 [5.4]	4 [4.1]
Solve disorientation/confusion	0 [0.0]	3 [14.3]	1 [2.7]	4 [4.1]
Get owner’s attention	1 [2.5]	1 [4.8]	1 [2.7]	3 [3.1]
Wake owner from nightmare	2 [5.0]	0 [0.0]	1 [2.7]	3 [3.1]
Keep owner focused	1 [2.5]	1 [4.8]	1 [2.7]	3 [3.1]
Provide excuse to leave	1 [2.5]	1 [4.8]	0 [0.0]	2 [2.0]
Others	1 [2.5]	1 [4.8]	3 [8.1]	5 [5.1]
Total number of dogs	40	21	37	98

**Table 7:** Numbers of service dogs performing specific tasks for medical support [%].

	Size			
	Small	Medium	Large	Total
**Specific tasks**	n [%]	n [%]	n [%]	n [%]
Alert to seizure	12 [52.2]	3 [85.3]	13 [32.5]	28 [40.0]
Alert for abnormal blood sugar level	1 [4.3]	1 [28.4]	13 [32.5]	15 [21.4]
Alert/waking up when irregular breathing	2 [8.7]	2 [56.9]	5 [12.5]	9 [12.9]
Seizure response	3 [13.0]	1 [28.4]	4 [10.0]	8 [11.4]
Alert for migraine	1 [4.3]	0 [0.0]	2 [5.0]	3 [4.3]
Pain control	3 [13.0]	0 [0.0]	0 [0.0]	3 [4.3]
Alert for choking	1 [4.3]	0 [0.0]	1 [2.5]	2 [2.9]
Alert for asthma	0 [0.0]	1 [28.4]	1 [2.5]	2 [2.9]
Others	3 [13.0]	0 [0.0]	5 [12.5]	8 [11.4]
Total number of dogs	23	7	40	70

### Changes in registrations over the years 1999–2012

The following analyses are based on the cumulative data (dataset 1) which included information on registrations for dogs over the 14 years.

#### Annual registrations: types of assistance or categories of work

Among 2,722 dogs for which information on the types of assistance dogs was available, hearing and guide dogs were registered at a rate of fewer than 30 dogs each year; but the number of service dogs registered rapidly escalated, reaching a peak of 382 registered service dogs in 2009 (0 dogs in 1999, 36 in 2000, 64 dogs in 2001 and in 2002, 71 in 2003, 96 in 2004, 97 in 2005, 207 in 2006, 201 in 2007, and 254 in 2008), and modest decreases then followed (346 in 2010, 335 in 2011, and 266 in 2012).

Concerning the categories of work for service dogs during this period of time, the proportions of dogs doing each type of work were: in the early three years (2000–2002, 76 dogs), mobility: 73.7% (56 dogs), psychiatric: 17.1% (13), medical: 9.2% (7), emotional: 0.0% (0); in the recent three years (2010–2012, 279 dogs), mobility: 36.9% (103 dogs), psychiatric: 31.9% (89), medical: 12.2% (34), emotional: 19.0% (53).

#### Annual registrations: uses of the main pure breeds

**Fig 2:**
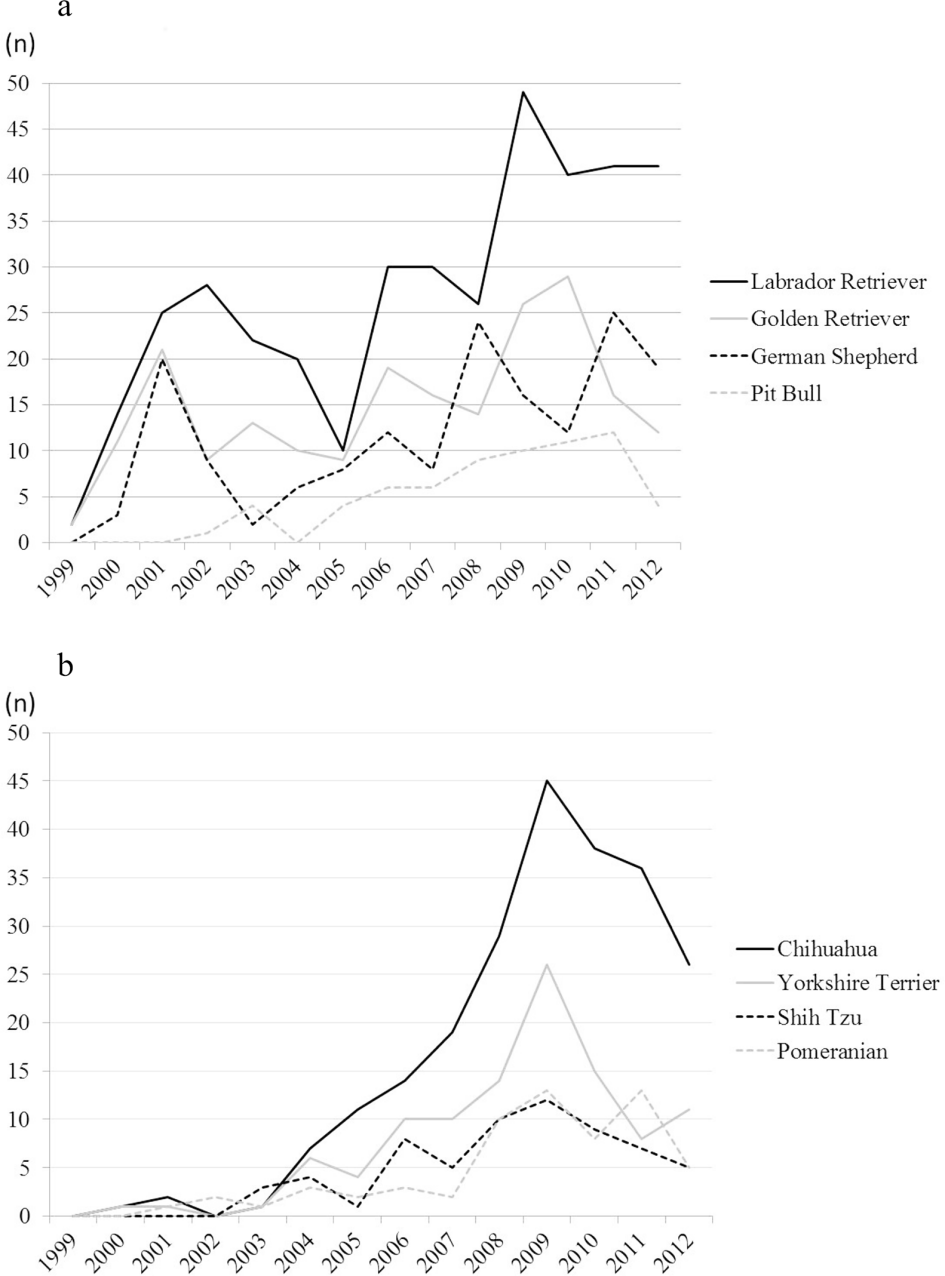
A) Most commonly registered large and medium dogs. Numbers of assistance dogs from the 4 most commonly registered breeds: categories of large and medium dogs from 1999 to 2012. B) Most commonly registered small dogs. Numbers of assistance dogs from the 4 most commonly registered breeds: category of small dogs from 1999 to 2012.

Fig 2A and 2B show the transitions for the top four pure breeds in their numbers among large and medium breeds (Fig 2A) and small breeds (Fig 2B). The large breeds were registered from the early years, but the small breeds initially were few and then increased gradually over the years up to 2009. Especially, the increase of Chihuahua was dramatic. But again, the numbers of small breeds registered slightly decreased after 2009.

#### Annual registrations: body sizes and categories of work

**Fig 3:**
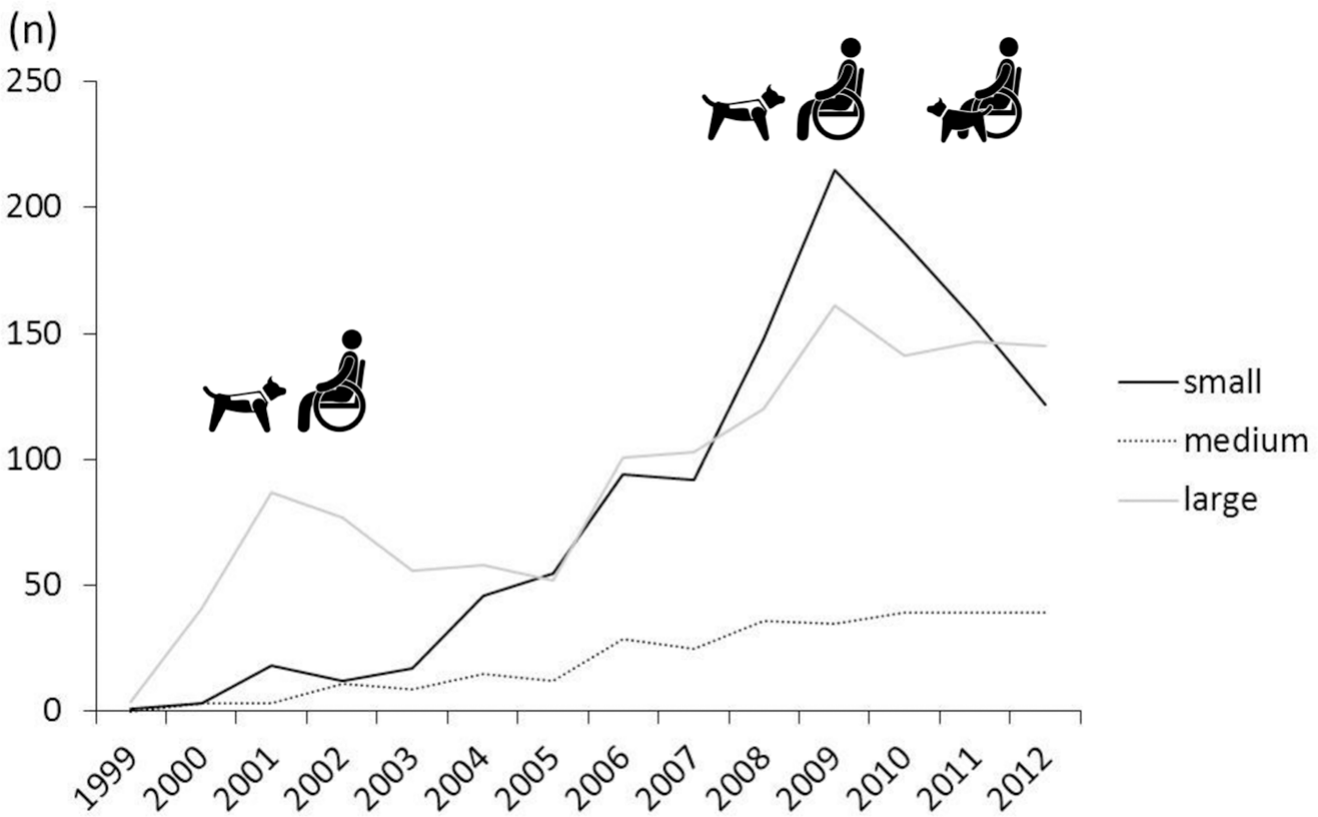
Numbers of assistance dogs registered: small, medium and large dogs from 1999–2012.

The body sizes were available from 2,752 registered dogs in dataset 1, as shown in Fig 3. The number of small dogs escalated from 1999 to 2009. By 2005, the number of small dogs registered equalled the number of large dogs, but it declined after 2009.

Table 8 shows the body sizes for each category of work for 2000–2002 and for 2010–2012. Over the 10 year interval, the proportion of small dogs increased in all categories of work.

**Table 8:** Numbers of dogs by body size for each category of work: early (2000–2002) and recent (2010–2012). [%]

		Small	Medium	Large
**Guide**	early	0 [0.0]	2 [3.4]	56 [96.6]
	recent	4 [11.8]	2 [5.9]	28 [82.4]
**Hearing**	early	6 [50.0]	0 [0.0]	6 [50.0]
	recent	13 [50.0]	4 [15.4]	9 [34.6]
**Mobility**	early	0 [0.0]	4 [8.0]	46 [92.0]
	recent	9 [13.0]	8 [11.6]	52 [75.4]
**Psychiatric**	early	4 [40.0]	2 [20.0]	4 [40.0]
	recent	30 [57.7]	8 [15.4]	14 [26.9]
**Medical**	early	1 [16.7]	1 [16.7]	4 [66.7]
	recent	5 [31.3]	0 [0.0]	11 [68.8]
**Multiple**	early	4 [12.1]	3 [9.1]	26 [78.8]
	recent	39 [31.0]	21 [16.7]	66 [52.4]
**Emotional**	early	0 [0.0]	0 [0.0]	0 [0.0]
	recent	12 [66.7]	2 [11.1]	4 [22.2]

## Discussion

### The numbers of registered dogs

Many dogs have been registered as assistance dogs in California. Considering only the 23 facilities providing records on active assistance dogs at the time of data collection, there were 2,796 registered assistance dogs. As an example of the proportion of licensed dogs that were registered, San Francisco had 1,627 registered assistance dogs, and 35,365 dogs were licensed at the date of December 31, 2012 (personal communication through SF government). Thus, 4.6% of licensed dogs in San Francisco were registered assistance dogs. Although California often is considered to have a liberal culture, and San Francisco has some major assistance dog organizations nearby, this proportion seems large. If the same ratio were used based on the number of pet dogs in the U.S. (69,926,000 dogs) [32], an estimated number of assistance dogs in the U.S. would be 3,216,550; this seems implausible. Additionally, there is no substantive method to confirm whether the handlers with dogs applying for the California ID tags satisfy the Code or not; a similar situation of claiming assistance dog status is happening throughout the U.S. It can be said that handlers claim many assistance dogs in the U.S. that may not satisfy the requirements that the U.S. DOJ established to fulfill the ADA.

The number of registered service dogs increased until 2009, and modestly decreased after that. Interests and demands for service dogs have grown in the society, plus the expansion may be related to the liberalized federal laws and regulations regarding assistance dogs and disabilities. Under the ADA and as specified in the U.S. DOJ implementing regulations, someone in a public accommodation is only allowed to ask if the animal is a service dog and what work or task the animal has been trained to perform; for non-evident disabilities, documentation from a health professional of the dog’s status for the person may be requested, but no evidence is required that the dog is a qualified assistance dog, nor any information on the nature of the person’s disability [18]. So long as the dog is individually trained to perform tasks supporting the disabilities of his/her human partner, the assistance dog partner can claim his/her own dog as an assistance dog, regardless of who trained the dog. No centralized U.S. process specifies a mandatory training program or registration or qualification system. Only if the dog wears a harness or a vest marked with a logo from a well-known assistance dog organization can one differentiate well-trained assistance dogs from perhaps-unqualified assistance dogs. The inconsistent nomenclature for assistance and service dogs across the federal and state laws is confusing, adding difficulty for people accompanying their assistance dogs, and people accepting those dogs in their facilities.

We found that ID tags were issued even for some dogs not considered as assistance dogs under the Food and Agriculture Code in California, nor the U.S. ADA and DOJ, such as therapy dogs, and many emotional support animals, including some cats. Although ESAs are not included in the California Food and Agriculture Code and the U.S. DOJ regulations, other federal laws from U.S. Housing and Urban Development (HUD) and Department of Transportation (DOT) allow the handlers of ESAs to accompany their pet animals to public transportation and housing and ESAs include other species [19, 20]. The ID tags may have been mistakenly issued to some cats because of the confusing inconsistent laws. Thus, even animal control facilities charged with registering California assistance dogs did not correctly understand the definitions of assistance dogs as specified in the laws. Furthermore, the ADA amendment act of 2008 clarified and broadened the definition of “disability” [21]. It defines “disability” as a physical or mental impairment that substantially limits one or more major life activities, which include but are not limited to caring for oneself, performing manual tasks, seeing, hearing, eating, sleeping, walking, standing, sitting, reaching, lifting, bending, speaking, breathing, learning, reading, concentrating, thinking, communicating, interacting with others, and working, and the operation of a major bodily function, including the operation of an individual organ within a body system. Registrations of assistance dogs increased the most from 2008 to 2009, suggesting that this broadened definition of disability may have encouraged people to think that they also had a qualifying disability and a right to use an assistance dog.

The rate of registrations of assistance dogs slowed after 2009, perhaps explained by a clarifying regulation from U.S. DOJ in 2010 which came into effect in March 2011 [18], as well as growing awareness of and concerns towards unqualified assistance dogs. The new DOJ regulation clearly specified that emotional support animals are not included as service dogs (assistance dogs). Concerns with people perhaps making fraudulent registrations of seizure alert or psychiatric service dogs have led some California facilities to inform the handlers that they might notify the Department of Motor Vehicles or DOJ because the health condition could affect the handler’s performance when driving or possessing firearms (personal communication with staff at a California animal control facility). The updated regulations by DOJ in 2010 and the concerns and efforts of some California animal control facilities may have affected the numbers of registrations and perhaps reduced incidents of falsely claimed assistance dogs. However, such people are still likely to bring their unqualified assistance dogs into public areas.

### Demographics of registered assistance dogs

The breeds, sizes, and ages of the registered assistance dogs were more diverse than those trained by the typical assistance dog training organizations. The conventional organizations breed and train specific breeds as guide and service dogs, and place the dogs with handlers when they are just a few years of age. By now, dogs from various sources are used by different training organizations in the U.S.; for example, some adopt potential assistance dogs from shelters or accept owners’ pet dogs to be trained as assistance dogs for the owners. Meanwhile, the ADI accredited organizations in the U.S. still commonly use large breeds such as Labrador Retrievers, Golden Retrievers, Standard Poodles, and mixed combinations of these breeds. We have no information on how many registered dogs were trained by the ADI accredited organizations or member organizations of the International Guide Dog Federation (IGDF). However, forty-eight ADI accredited programs place assistance dogs in California, plus there are three guide dog schools which are members of the IGDF. Therefore, many dogs trained by those organizations are placed in California. Nonetheless, our data showed diverse breeds, body sizes, and ages, suggesting that many of these assistance dogs registered in California were trained by the person with disabilities or private dog trainers rather than by the typical assistance dog organizations. Handlers lacking the backing of a well-known assistance dog organization may view the ID tag issued by an animal control facility as a secure tool to label their dogs as assistance dogs.

The large breeds mentioned above are often preferred as assistance dogs because of their useful characteristics, including their temperament, trainability, and body size [33–35]. Yet, Chihuahua was the second most registered breed in California after Labrador Retriever, and Yorkshire Terrier was the fifth most common, after German Shepherd and Golden Retriever. According to Hart and Hart [36], the small breeds such as Chihuahua and the terriers are rated as having lower trainability and excessive barking, traits that seem less adequate for assistance dogs. Furthermore, the Pit Bull type was commonly registered, even though there are some areas of the U.S. where Pit Bulls are banned or restricted due to being considered a dangerous breed; of course they are often kept as companion dogs in the U.S. [37]. When the animal control facilities issue ID tags there are no restrictions for breeds, so one expects registrations of the popular breeds.

Also, initial registrations of smaller dogs at older ages were more common than for dogs having large body sizes. These registered assistance dogs included many dogs which were not chosen at young ages nor bred to become assistance dogs. Perhaps they had good temperaments for being assistance dogs and had been adopted from shelters, or the owners may have had certain reasons for choosing their breeds or realized innate abilities of their pet dogs to assist with their disabilities and trained them as assistance dogs. Especially for the last case mentioned, the convenience of taking care of a dog, or even just a preference for a certain breed, would have been more important factors for handlers in choosing their dogs, rather than the dogs’ traits for assisting, such as trainability, temperament, and body size.

Concerning ages of the dogs, 478 dogs were registered at an age older than 10 years. Dogs may learn new things at any ages, but Adams and her colleagues indicated that spatial learning and memory is sensitive to age in the dog: aged dogs (8–12 years old) show impaired spatial learning and display spatial working memory deficits compared to young dogs (1–3 years old) [38]. Also, compared to young (2.91–3.73 years old) and middle-aged (4.05–5.50 years old) dogs, old (8.61–10.94 years old) and senior (11.10–13.81 years old) dogs were impaired on the initial learning of the tasks conducted in one study [39]. Training dogs at older ages as assistance dogs seems inappropriate in terms of its efficiency and the working life of the dog. Further, older dogs experience arthritis, and visual and hearing disabilities. Therefore, assistance dogs trained by organizations usually are retired around 10 or 11 years of age [40, 41]. Considering their health, longevity, and efficiency for the training, it is unrealistic to register dogs as assistance dogs after they are 10 years of age.

### Dogs’ assisting tasks and body sizes

In recent years, the proportions of service dogs registered for psychiatric, medical, or emotional support came to exceed those registered for mobility support, which had been the main work for service dogs 10 years ago. Thus, new types of service dogs came into use and they now are more numerous than the traditional service dogs assisting with mobility, guiding, and hearing support.

Dogs with small body sizes were mainly used as hearing dogs and service dogs for psychiatric or emotional support: these dogs have less need for height or strength than guide dogs and service dogs providing mobility support. However, the dogs’ body sizes shifted toward smaller across the 10 years for all types of assistance dogs. Among guide dogs, 10% (4 dogs) of those registered in the most recent three years were small dogs, less than 11 inches in height. Guide dog handlers conventionally receive information from their guide dogs through the harness and U-shaped handle, and they are required to disobey unsafe commands [42]. Considering conventional uses of guide dogs, it seems unrealistic to use such small dogs; perhaps they were falsely claimed as guide dogs. However, in the old illustrations report by Fishman about the evolution of the use of guide dogs, some blind people were guided by small dogs [3]. Therefore, the registered small dogs could have guided their owners in a unique way which was invented in their relationship.

Among the service dogs, large dogs were used primarily for mobility support. They performed a variety of tasks, whereas small and medium dogs were mainly used for retrieving objects. But some small dogs performed tasks such as balancing, opening/closing doors, pulling and pushing wheelchairs, and supporting the handler to stand up. Coppinger and his colleagues reported that undue stress was placed on service dogs when they performed tasks such as opening doors and pulling a wheelchair, even for large dogs [43]. Details on the assisting procedures were not provided, but if the small dogs were used for these tasks in the same way as the large dogs perform them, it could burden the body of small dogs. Such uses of dogs are questionable when considering animal welfare.

There were no general body size characteristics for psychiatric service dogs performing various tasks. But more small and medium sized dogs performed tasks to alleviate symptoms such as anxiety, depression, panic and stress compared to large dogs. For medical service dogs, medium and large dogs were used more often for alerting to abnormal blood sugar levels. On the other hand, only small dogs were used for pain control. Small dogs might be suitable to perform tasks related to cuddling up to the handlers.

Dogs with different body sizes were used for a great variety of tasks. Therefore, one could not estimate what tasks the dog performed from the appearance or body size of the dog. Also, a third party could not determine whether the dog is an adequately trained service dog just from ascertaining the breed or breed type.

The obvious surge in popularity of small dogs for assistance may also relate to the convenience and ease of handling a smaller dog. People living in apartments may appreciate a dog taking up less space. Less human strength is required when managing a small dog, which accommodates older persons and certain disabilities. For tasks not requiring a dog with considerable strength or size, people may be preferring the smaller body size.

### Welfare of assistance dogs

Considering the welfare of working dogs is important for their sustainable future [44], and there are some studies on the welfare of assistance dogs [43, 45]. Sources of stress for some assistance dogs included poor instruction from the handlers [43] and lack of social play and rest [45]. These reports indicated that handlers of assistance dogs have to understand how to handle dogs and the dogs’ physical and psychological requirements. Moreover, choosing dogs suited for working as assistance dogs is essential, including not only whether the dogs are trainable to perform the required tasks, but also their temperaments. More stimuli are in the working environments of assistance dogs than in the environments of pet dogs. Dogs that are sensitive to new environments could be stressed working as assistance dogs. Some reports show that chronic and acute stress can cause physiological changes, such as increased heart rates and cortisol levels [46, 47]. Assistance dog training organizations consider the temperaments and aptitudes of dogs as they place each assistance dog; they breed their dogs and/or evaluate and select dogs when they recruit dogs from other sources [34, 48]. People choosing to train their own pet dogs for assistance need to consider the dog and whether it is appropriate from the various aspects, such as their temperaments, behaviors, and burdens on their bodies and mental health.

### Unqualified assistance dogs and regulations

Although applicants signed affidavits and ID tags were issued from the California animal control facilities, many non-assistance dogs were inappropriately registered. This is one example showing that in the U.S., many dogs that do not legally qualify for status as assistance dogs are brought into public areas.

The increased numbers of unqualified assistance dogs may lead to problems such as increased rejection of public access for people with legitimately trained assistance dogs, perhaps especially when privately trained assistance dogs of uncommon breeds are involved. The use of unqualified assistance dogs also can impact public safety and increase dog bites or attacks on other dogs. Inappropriate behaviors or poor hygiene of dogs are also problems, especially for people who are afraid of dogs and have allergies to them. The welfare of dogs is at risk and can lead to poor physical and mental health of these and other dogs. If a U.S. mechanism were established for screening out dogs that are inappropriately presented as assistance dogs and certifying legitimately trained assistance dogs, the number of unqualified assistance dogs could be reduced. This would benefit society, assistance dog handlers, and the dogs themselves in terms of the assistance dogs being welcomed in a wide range of places and having better welfare. Some countries assure the quality of assistance dogs by their regulations.

In Japan, in addition to the guide dogs which are certified by each qualified training organization, hearing dogs and service dogs are certified only by specific organizations designated by the Minister of Health, Labour and Welfare. The certified assistance dog teams are required to carry documentation, including the certificate and the records of the dogs’ healthcare; these have to be offered when requested [26]. Showing such documentation protects the person’s privacy concerning their disabilities, rather than asking the person what tasks the dog is trained to perform to support the person’s disabilities, as allowed in U.S. legislation. Japanese certification requires a comprehensive examination of the assistance dog and the handler by the multidisciplinary team, including the dog trainer, physician, veterinarian, social worker, and physical therapist, and an occupational therapist for the service dog team, and a speech therapist for the hearing dog team. Therefore, the behavior and performance of the assistance dogs, appropriateness of the handler using the assistance dog, and the health of the dogs are screened; this may help to avoid undue physical stress for the dogs.

Similarly, assistance dogs are certified by the specified or approved organizations or trainers in New Zealand and some parts of Australia like Queensland, respectively [13, 14]. On the other hand, the European countries follow the standards of the International Guide Dog Federation (IGDF) and the Assistance Dogs International (ADI) concerning regulations related to the public access of people accompanying an assistance dog. For example, in the European Union (EU) member states, the EU Regulation (EC) No.1107/2006 requires that air carriers shall accept the carriage of “recognised assistance dogs” in the cabin, subject to national regulations [15] and also requires that the “recognised assistance dogs” are trained by an organization that is accepted by and affiliated to the IGDF or that meets the full membership criteria of the ADI [17].

The U.S. offers an advantage that people can live with any kind of assistance dog; this has led to legally creating new tasks and roles for assistance dogs so long as the regulations are met. While retaining the U.S. flexibility that allows dogs to address a wide range of disabilities, some enforcement is needed to assure the safety of society and the welfare of the assistance dogs, as well as supporting the people with disabilities in living with a properly trained assistance dog.

One limitation of this study is that only half of the animal control facilities which issued Assistance Dog Identification tags disclosed the information for this study. The rest of the facilities issuing tags might have a different demographic of assistance dogs but we could not investigate them further. Another limitation of this study is that the collected information on the dogs was self-reported by the applicants. The date and general description of the dog at registration would seem likely to be accurate, but the accuracy of the handlers’ specific descriptions of the dogs’ breeds and tasks are unknown. Also, we were able to collect the more detailed information only from a limited portion of the registered dogs, as each animal control facility gathered somewhat different types of information. The available information gave little explanation on the training history of registered dogs. Therefore, we cannot conclude that the dogs with unique breeds, sizes, and ages compared to the traditional assistance dogs were unqualified dogs; some of them may have been properly trained. Further, in the liberal state of California where there are several pioneering training facilities for assistance dogs, people may have more positive attitudes towards them. Therefore, we cannot generalize all results reported in this study to other parts of the U.S.

In conclusion, we found that the numbers of assistance dogs, especially service dogs, sharply increased in the past decade in California. Dogs with small body sizes, and new types of service dogs, such as service dogs for psychiatric and medical assistance, mainly contributed to the increase. In addition, the Assistance Dog Identification tags were mistakenly issued to some dogs which do not fall within the definition of assistance dogs under the law, such as emotional support animals. This indicates that the state governmental registering system does not work properly. Also, a substantive number of ID tags were issued to dogs that seem not appropriate to use as assistance dogs, such as old dogs which were registered for the first time when they were 10 years or older.

No research data are available showing the current situation of assistance dogs in the U.S., but this study provided evidence of a prevailing misuse and misunderstanding of assistance dogs in California. The results point to the usefulness of establishing a mechanism to qualify and certify assistance dogs which are trained properly and legitimately.
